# Friction-Wear Characteristics of Carbon Fiber Reinforced Paper-Based Friction Materials under Different Working Conditions

**DOI:** 10.3390/ma15103682

**Published:** 2022-05-20

**Authors:** Zhiwei Ma, Changsong Zheng, Cenbo Xiong, Liang Yu, Yujian Liu, Cunzheng Zhang

**Affiliations:** School of Mechanical Engineering, Beijing Institute of Technology, Beijing 100081, China; 3220200292@bit.edu.cn (Z.M.); zhengchangsong@bit.edu.cn (C.Z.); yuliang@bit.edu.cn (L.Y.); 3220200280@bit.edu.cn (Y.L.); 3220190365@bit.edu.cn (C.Z.)

**Keywords:** paper-based friction materials, carbon fibers, SAE#2 test bench

## Abstract

To study the friction and wear performance of carbon fiber reinforced friction materials under different working conditions, paper-based friction materials with different fibers were prepared. Experiments on the SAE#2 test bench were conducted to study the infectors including friction torques, surface temperature, coefficient of friction (COF), and surface morphologies. The results were analyzed, which indicated that the carbon fiber reinforced friction material could provide a higher friction torque and a lower temperature rising rate under the applied high pressure and high rotating speed conditions. As the pressure increased from 1 MPa to 2.5 MPa, the friction torque of plant fiber reinforced material increased by 150%, the friction torque of carbon fiber reinforced material increased by 400%, and the maximum temperature of plant fiber reinforced and carbon fiber reinforced material reached the highest value at 1.5 MPa. Thus, carbon fibers not only improved the COF and friction torque performance but also had advantages in avoiding thermal failure. Meanwhile, carbon fiber reinforced friction materials can provide a more stable COF as its variable coefficient (α) only rose from 38.18 to 264.62, from 1 MPa to 2.5 MPa, which was much lower than the natural fiber reinforced friction materials. Simultaneously, due to the good dispersion and excellent mechanical properties of PAN chopped carbon fibers, fewer pores formed on the initial surface, which improved the high wear resistance, especially in the intermedia disc.

## 1. Introduction

In the wet multi-plate clutch, the satisfactory performance of the friction material directly determines the application range and lifetime of the clutch [1,2,3]. With the development of the wet multi-plate clutch towards high power density, low energy consumption, and no environmental pollution, the requirements of paper-based friction materials are continuously being improved [4,5,6,7], especially in terms of friction-wear performance, mechanical strength, heat resistance, load adaptability, and environment-friendly performance [8,9]. Paper-based friction materials consist of fillers, fibers, binders, and modifiers, which are generally selected and mixed by trial-and-error method [10,11,12]. Therefore, the friction and wear performance of paper-based friction material mainly depends on the inherent physical and chemical properties of the integrated material [13,14]. However, various factors affect the friction and wear properties of paper-based friction materials, such as applied pressure, rotating speed, ATF temperature, and flow rate of lubrication [15,16]. Research aimed at achieving these higher requirements has recently attracted much attention. Reinforcing fibers have gradually evolved from the initial asbestos fibers and natural fibers, to more recently, the current glass fibers, Kevlar fibers, and carbon fibers [10,12,17].

Asbestos fibers are prone to producing carcinogens at high temperatures, which affect human health, and are gradually being banned [10]. Natural fibers such as sisal, flax, jute, hemp, coir, banana, coconut powder, pineapple and date palm have been the basis of recent experimental study [18]. Due to sufficient raw materials, simple production, environmental protection, safety, and no environmental pollution, natural fibers have long been used as a replacement material for asbestos fiber [19,20,21]. Tej Singh [22] developed automotive brake friction composites containing 5, 10, 15, and 20 weight percentages of natural fibers (hemp, ramie, and pineapple) that were analyzed for tribological properties. The result showed that the performance coefficient of friction (0.548) and friction-stability (0.93) remained highest for 5 wt% pineapple fiber composites. The application of glass fiber in friction materials has also been largely developed due to its good heat resistance and mechanical properties [23]. Due to the increased power density of the clutch and its more complex use environment, the requirements for mechanical properties such as strength are constantly increasing, and other reinforcing fibers need to be developed to improve friction and wear performance. Researchers have made many attempts, including glass fibers and carbon fibers.

Xiang Zhang [24] studied the effect of glass fiber on the tribological properties of composite friction material. The results showed that the friction coefficient (μ_d_) of the sample with 10 wt% glass fiber was higher, and the wear rate was lower. The shear strength initially increased and then decreased, but the compressibility increased and the recovery decreased as the glass fiber content increased. Jae Hyun Gweon [23] studied the friction and wear characteristics of chopped glass fibers on the material. The results showed that the uniform distribution of glass fibers produced a smoother material surface, resulting in stronger contact hardness and a higher friction coefficient. With the development of new fiber types and the maturity of processing technology, carbon fiber is gradually being popularized in instructional applications [25,26,27] because it has high specific strength, excellent mechanical properties, and certain self-lubricating properties. Jie Fei [26,28] prepared four kinds of micron-level carbon fiber reinforced paper-based friction materials and experiments on a plate-on-plate wet friction performance tester to study the effects of fiber content and phenolic resin content on the performance of friction materials. The results showed that the wear rate of samples increased with the carbon fiber content, and a type of continuous friction film was formed on the work surface with the increasing resin content, which efficiently enhanced the wear resistance of the samples. The researchers mainly focused on the effect of the carbon fiber content, and did not quantitatively explore and evaluate the effect of carbon fiber on friction and wear performance.

This paper focuses on the comparison of the friction and wear performance between natural fiber and carbon fiber reinforced materials. The effect of carbon fiber on the friction and wear properties of paper-based material under different working conditions was studied systematically, and the improvement was described quantitatively. The research results provide a theoretical basis for the improvement of paper-based friction materials and the application of carbon fibers.

## 2. Materials and Methods

### 2.1. Testing Equipment

**Figure 1:**
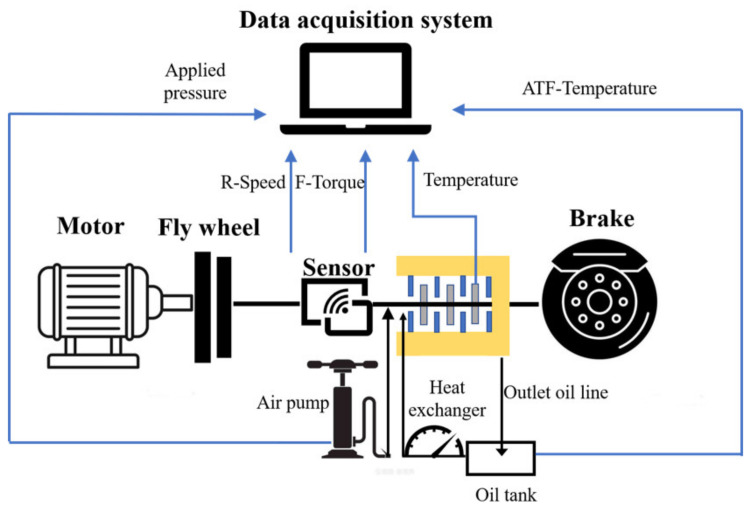
Schematic diagram of the SAE#2 clutch test bench. R-Speed is rotating speed and F-Torque is friction torque.

The pin-on-disk test is often used as an important means of studying friction and wear characteristics [29]. To highlight the practical application scenarios of the research, the SAE#2 bench test was used in this paper to study the practicability of friction materials in the wet clutch. The SAE#2 test was released by the American Society of Mechanical Engineering (New York, NY, USA) in 2000. It is mainly used to evaluate the friction performance of an automobile transmission disc clutch under complex and changeable actual working conditions using standard J2490. The test bench is shown in Figure 1, which includes a flywheel, clutch pack, brake, lubrication system, heat exchanger, data acquisition system (Hangzhou, China), etc. The lubrication system can cool and lubricate the friction components, the heat exchanger is responsible for controlling the inlet ATF temperature, and the data acquisition system collects all relevant dynamic signals. The main parameters of operation conditions include ATF temperature, applied pressure, and rotating speed, controlled by the operating system. The initial temperature of ATF flow was maintained at 60, 80, 100, and 120 °C, respectively. At the same time, the flow rate was kept constant at 4 × 10^−3^ L/(min·m^2^). The applied pressures were 1, 1.5, 2 and 2.5 MPa which were applied by the air pump through compressed air. To study its working performance under conditions of low and high rotating speeds, the rotating speeds were 600, 1000, 1500, and 2700 rpm. The specific working parameters are shown in Table 1.

The surface morphology of the friction plate before and after the test is an important parameter to analyze its friction mechanism and wear degree. A scanning electron microscope (SEM) (JEOL, Tokyo, Japan) was used to observe the surface topography of the friction discs.

### 2.2. Test Samples

**Figure 2:**
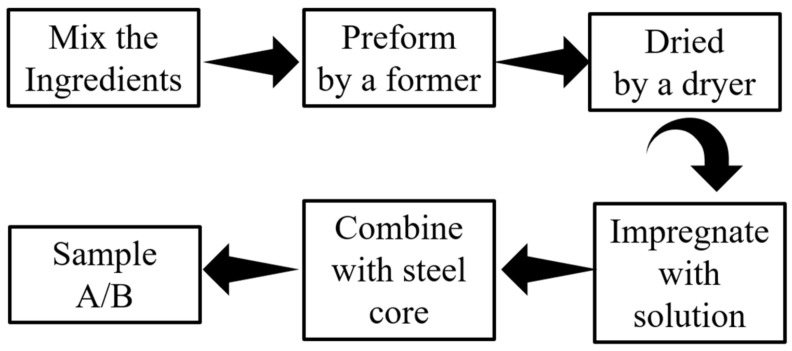
Manufacturing process of sample A/B.

To study the effect of carbon fiber on friction materials under different working conditions, the choice of raw ingredients is crucial. In this experiment, PAN chopped carbon fibers and plant fibers were selected as reinforcing fibers for two different paper-based friction materials. PAN chopped carbon fiber has a simple and mature manufacturing process, excellent physical and chemical properties and good dispersion properties. The most widely used plant fiber was selected as the reinforcing fiber of the paper-based friction material, which has a simple production process and a long history of use in paper-based material. The carbon fiber reinforced and natural fiber reinforced paper-based friction materials were designed and manufactured by the same manufacturing process. The slurry was prepared using a lab mixer to mix the plant fiber, friction performance modifier, filler, and water, in certain proportions. The slurry was processed into the preform by the former, which was dried by a dryer for a certain number of minutes. The preform was impregnated with a modified phenolic resin ethanol solution and shaped at a certain temperature for a certain period. The nature fiber reinforced friction materials were combined with the steel core plate to form sample A. The natural fibers were replaced with carbon fiber of length 3–12 mm to obtain sample B. The production process of the samples is shown in Figure 2.

**Figure 3:**
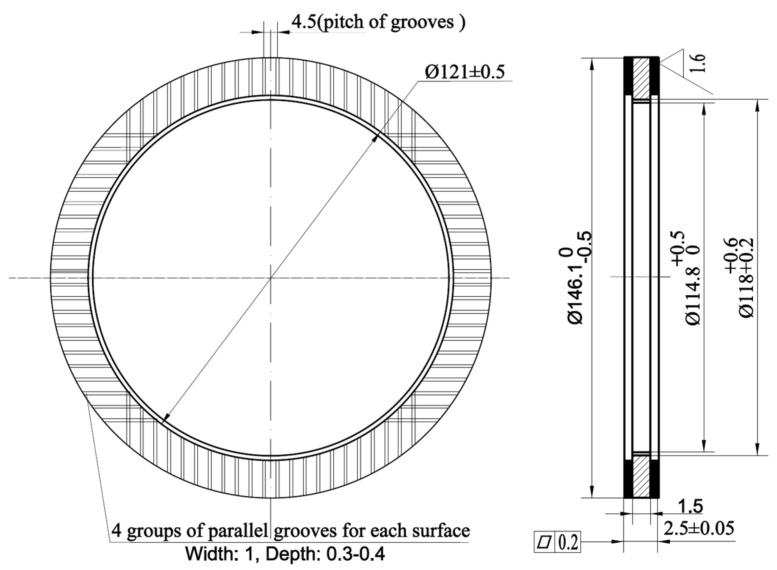
Schematic of the test samples.

**Figure 4:**
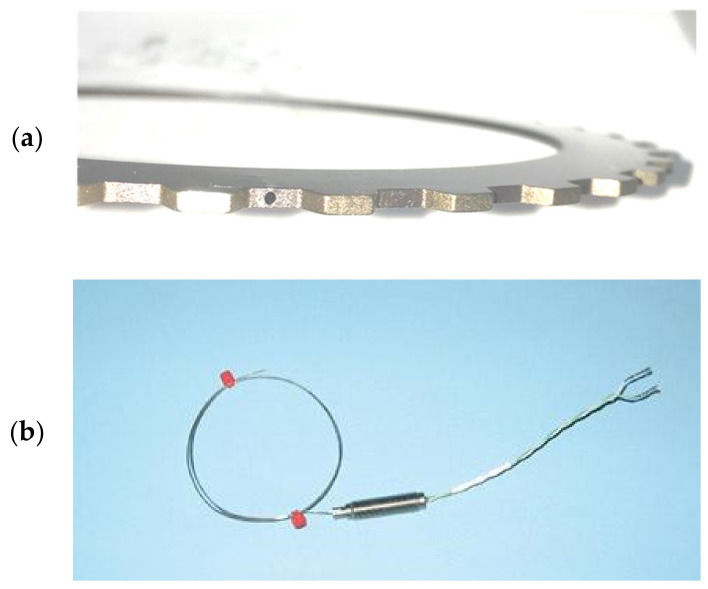
Thermometer hole of the samples (**a**), and the thermocouples (**b**).

The inner and outer diameters of the samples are shown in Figure 3, which had an outer radius of 73 mm and an inner radius of 60 mm. The machine parallel grooves on the surface of the friction plate guided ATF. The groove was 0.3 to 0.4 mm in depth and 1.0 mm in width, its total area accounting for 50% of the nominal area of the friction surface. At the time of the experiment, the temperature of the friction plate could not be directly measured, and the friction plate was closely attached to the steel plate, so the temperature of the steel plate was used to express the temperature of the friction plate. To measure the temperature of the steel plate, a k-type thermocouple was glued into its mid-plane, and the depth of the thermometer hole was 6 mm, as shown in Figure 4.

### 2.3. Testing Method

**Figure 5:**
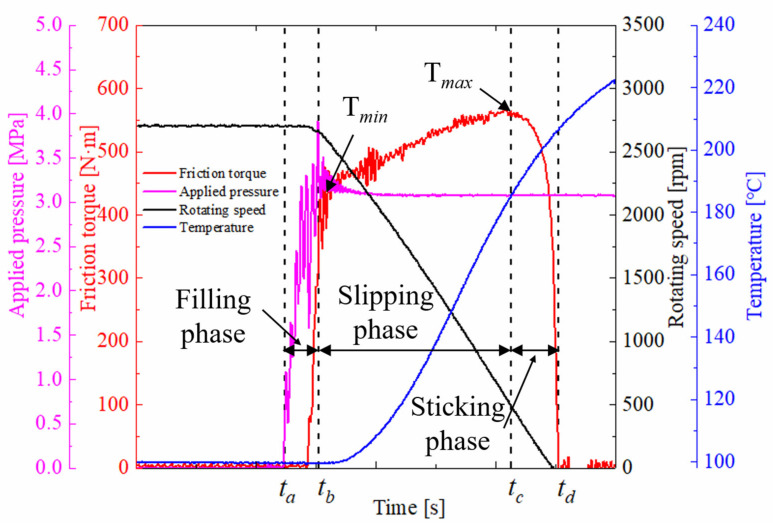
Representative measurement signals.

The SAE#2 bench experiments were in full compliance with the test standard of SAE J2490. Before the experiments, a run-in test was carried out for 200 duty cycles to stabilize the friction surfaces. Figure 5 shows the representative measurement signals in one duty cycle. The test steps were as follows. Firstly, the heat exchanger started to heat (or cool) the inlet ATF until the temperature requirements were reached. Then, the motor began to rotate with the flywheel and the clutch driving part. When they reached the pre-defined speed, the motor powered off automatically, and the applied pressure was exerted on the clutch piston at a time instant, t_a_. Then, the pressurized piston pushed the friction components moving in the axial direction until the gap between the friction components was eliminated. This process occurred during the filling phase between time instants t_a_ and t_b_. As the applied pressure increased, the friction contact between the friction components gradually became dominant, contributing to an increase in the friction torque and the decline of the rotating speed. The applied pressure reached the pre-defined value at time instant t_b_, where the friction torque was set to T_min_. Subsequently, the clutch was in the slipping phase, from t_b_ to t_c_. At time instant t_c_, the friction torque reached the maximum value T_max_. Finally, the clutch engagement was performed and entered the sticking phase when the rotating speed fell to nil at the lock-up time, t_d_.

As seen in Table 1, the experimental conditions were divided into three parts including 4 kinds of signals that corresponded to a cycle duty. Before the experiments, the surface temperature of the friction components was the same as the ATF temperature. The experiment for each duty circle was continuously repeated 5 times, and the data acquisition system randomly stored one set of the experimental data as the final results.

The contact pressure was assumed to be uniformly distributed over the friction surface, so the instantaneous COF *μ*(*τ*) of the clutch can be given as:

(1) $$\mu \left(\tau \right)=\frac{3T(\tau )\left(R_{o}^{2}-R_{i}^{2}\right)}{2zF_{app}(\tau )\left(R_{o}^{3}-R_{i}^{3}\right)}$$

where *z* is the number of friction pairs, *T* is the friction torque, *F_app_* is the axial force on the friction surface (N), and *R_i_* and *R_o_* are the inner and outer radii (mm), respectively.

The mean value of the instantaneous COF is primarily important to evaluate the frictional performance. Thus, the mean COF can be calculated via the variation of friction torque at the slipping phase as follows:

(2) $$\mu_{a}=\frac{1}{t_{d}-t_{b}}\int_{t_{b}}^{t_{d}}\mu (\tau )\;d\tau$$

The changes in instantaneous COF during the engagement process cannot be described by the mean COF. The variable coefficient is used to describe the variation of the instantaneous COF. The smaller the variable coefficient is, the better the friction stability is. It can be given as follows:

(3) $$\alpha =\frac{\mu \left(t_{c}\right)-\mu \left(t_{b}\right)}{t_{c}-t_{b}}$$

where *μ*(*t_c_*) and *μ*(*t_b_*) are instantaneous COF at *t_c_* and *t_b_*, respectively.

## 3. Results and Discussion

### 3.1. The Influence Factors of Friction Performance

#### 3.1.1. Applied Pressure

**Figure 6:**
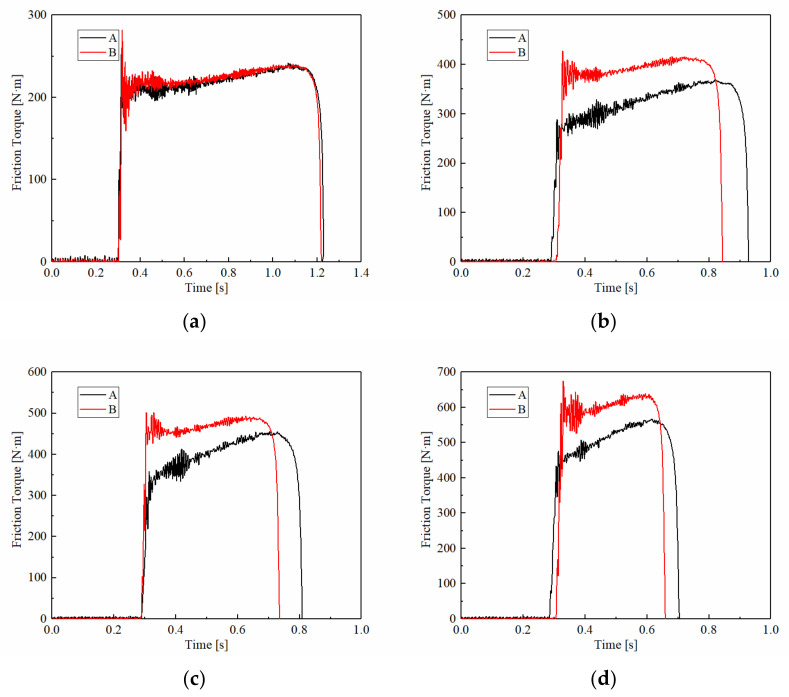
Variations of the friction torque at different applied pressures. (**a**) 1 MPa; (**b**) 1.5 MPa; (**c**) 2 MPa; (**d**) 2.5 MPa.

Figure 6 demonstrates the variations of the friction torque under different applied pressures. The friction torques of A and B both remained around 200 N·m at 1 MPa and rarely had differences in the torque curve, as shown in Figure 6a. With increasing pressure, the friction torque increased significantly. Under the pressure of 2.5 MPa, the mean friction torque of A was around 500 N·m which increased by 150%, and the mean friction torque of B was 600 N·m which increased by 400%. Overall, it can be concluded that the friction torque increased significantly with increasing applied pressure. This phenomenon can be explained as follows. In the duty cycle, the kinetic energy is converted into heat energy, which is dissipated into the air through the lubricating cooling system to maintain the working stability of the friction material. Therefore, the kinetic energy and heat dissipation performance is consistent under the same working conditions. The increase in pressure leads to more surface asperity contact and a larger effective contact area on a macroscopic scale, which leads to a larger friction torque.

However, the T_min_ of sample A increased from 200 N·m at 1 MPa to 450 N·m at 2.5 MPa, while sample B increased from 400 N·m to 600 N·m. The T_min_ of carbon fiber reinforced materials was 30% to 100% higher than materials with natural fibers. Similarly, the T_max_s maintained the same rules, which for sample A increased from 250 N·m at 1 MPa to 550 N·m at 2.5 MPa, and for sample B increased from 250 N·m to 650 N·m. However, the friction torque curves of sample A were much steeper than sample B. The variable coefficient under 2.5 MPa of A was 483.4, and for sample B was 263.5, implying that the friction stability of B was higher than A.

**Figure 7:**
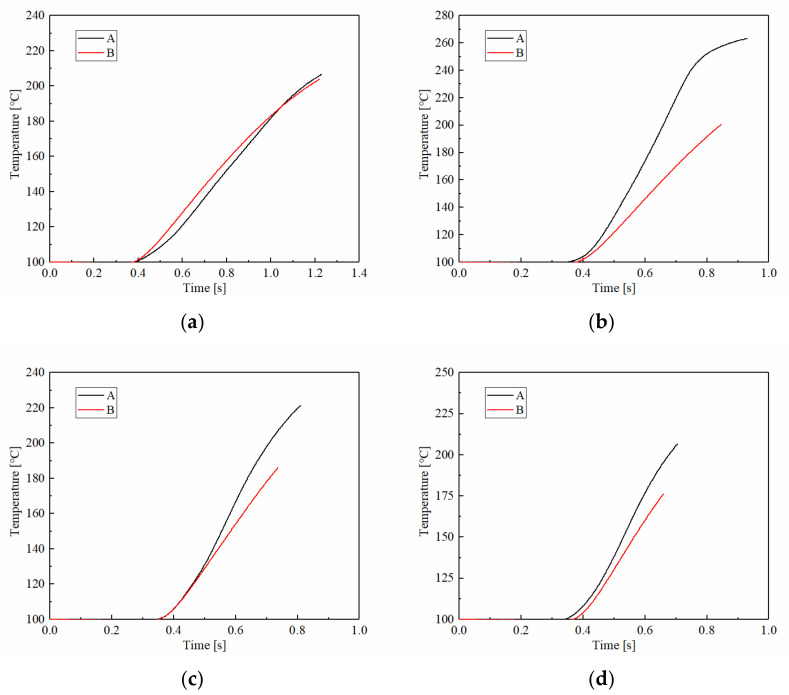
The temperature changes of samples A and B at different applied pressures. (**a**) 1 MPa; (**b**) 1.5 MPa; (**c**) 2 MPa; (**d**) 2.5 MPa.

At the same time, the applied pressure also causes the temperature to rise faster. The temperature changes of samples A and B under different applied pressures are shown in Figure 7. As for the changes of friction torque, the temperature rise curves of A and B were the same; both reached the maximum temperature of around 210 °C at 1 MPa. However, with the increase in applied pressure, the temperature changes were significantly different. The temperature rising rate and the maximum temperature of A were higher than B at 1.5 MPa, where it reached the highest temperature. For example, the maximum temperature of A was 263 °C approximately at 1.5 MPa, and B, eventually, was only 200 °C. Meanwhile, the maximum temperatures of A and B under 2.5 MPa were 206 °C and 175 °C, respectively. This showed that as the applied pressure continued to increase, the maximum temperature decreased gradually, and the difference in maximum temperature between A and B also reduced slightly. This is because maximum temperature is related to applied pressure and engagement time. With the applied pressure increasing, the engagement time decreased significantly. It resulted in the maximum temperatures of A and B reaching their highest values at 1.5 MPa, and then gradually decreasing with the increase in applied pressure. In addition, the engagement time of B was shorter than A, at 0.929 s and 0.846 s at 1.5 MPa, respectively.

#### 3.1.2. Rotating Speed

**Figure 8:**
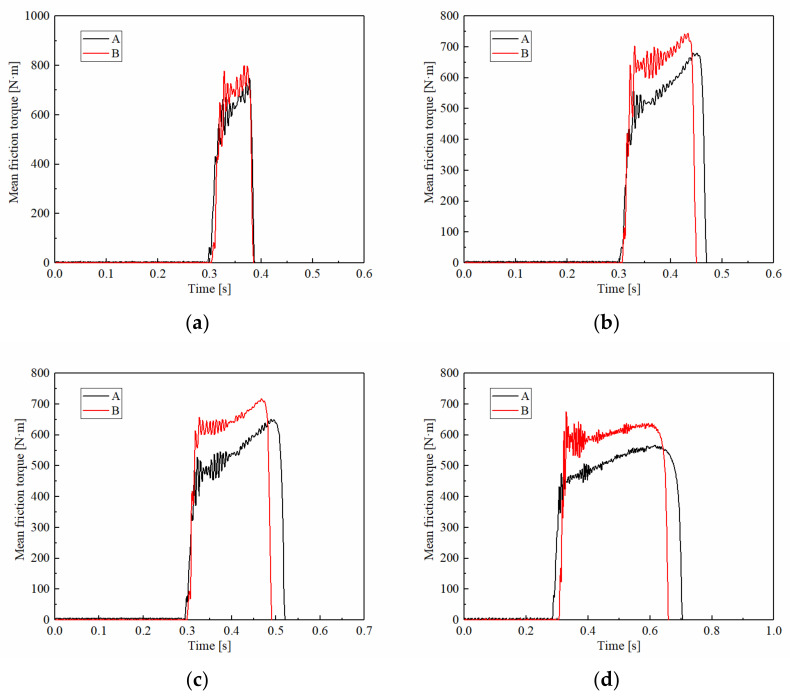
Variations of the friction torque at different rotating speeds. (**a**) 600 rpm; (**b**) 1000 rpm; (**c**) 1500 rpm; (**d**) 2700 rpm.

The friction performances of A and B were different under the rotating speed conditions. Figure 8 shows the curves of the friction torque at different rotating speeds, with the ATF temperature at 100 °C and the applied pressure at 2.5 MPa. The friction torque decreased with rotating speed. As the rotating speed increased from 600 rpm to 2700 rpm, the T_min_ of A decreased from 552 N·m to 413 N·m. Sample B showed the same phenomenon, as the T_min_ of B decreased from 670 N·m to 553 N·m. At the same time, the difference in T_min_ between the A and B changed slightly, only rising from 122 N·m at 600 rpm to 140 N·m at 2700rpm. The variation of T_max_ was similar to T_min_. However, the variable coefficient of A was much higher than B, which at 1500 rpm, was 1330 and 655, respectively. The changes showed that the friction torque increased slightly with rotating speed, and the friction torque stability of B was indeed better than A.

**Figure 9:**
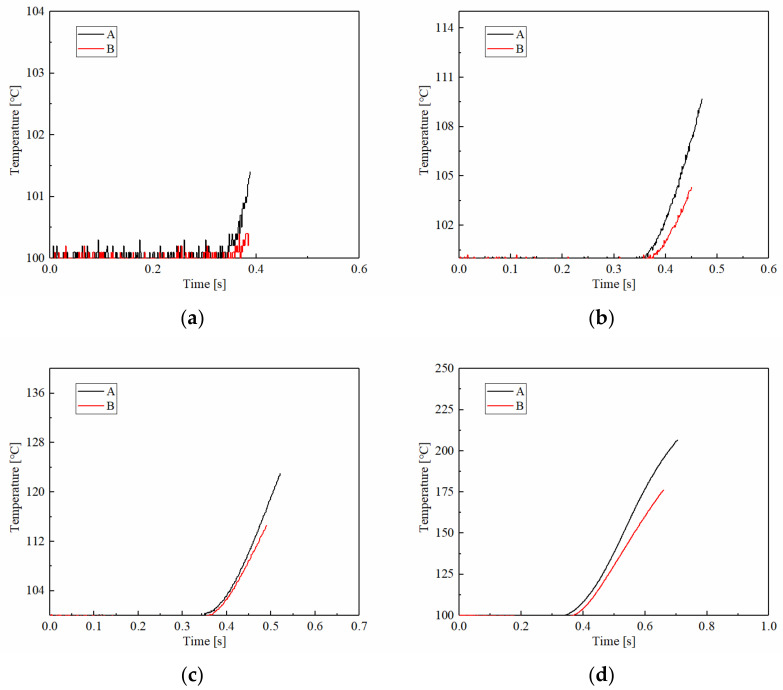
Temperature changes of samples A and B at different rotating speeds. (**a**) 600 rpm; (**b**) 1000 rpm; (**c**) 1500 rpm; (**d**) 2700 rpm.

The temperature changes are shown in Figure 9. As the rotating speed increased, the maximum temperature of the friction plate also clearly increased. The temperature of A rose from 101.5 °C at 600 rpm, to 206.6 °C at 2700 rpm. Under the same conditions, the maximum temperature of B rose from 100 °C to 176 °C. The temperature rising rate, which is the slope of the temperature curve, of A, was also significantly higher than B. It showed that B could provide a higher friction torque while maintaining a lower temperature under the high rotating speed condition. This is because carbon fiber has higher thermal conductivity and better cooling properties than natural fibers. Even carbon fibers with a high modulus have higher thermal conductivity than aluminum alloys.

#### 3.1.3. ATF Temperature

**Figure 10:**
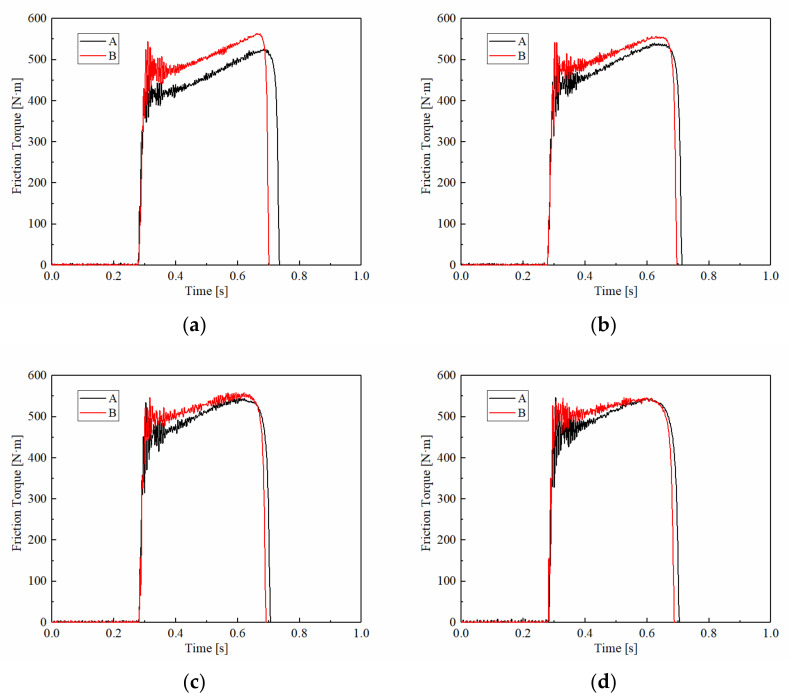
Variations of the friction torque at different ATF temperatures: (**a**) 60 °C; (**b**) 80 °C; (**c**) 100 °C; (**d**) 120 °C.

The changing trends of friction torque under different ATF temperature conditions were different from the other conditions. Figure 10 reveals the friction torque curves of A and B at different initial ATF temperatures. The T_max_ of A reached 521 N·m at 60 °C, while B reached 561 N·m at the same ATF temperature. As the ATF temperature increased, the T_max_ did not change significantly, and the T_max_ of A and B were the same at 120 °C, which was 540 N·m. The T_min_ of A was lower than that of B, but it did not change with the conditions. It can be regarded as a parameter which is insensitive to ATF temperature. The variable coefficient of A increased from 363 at 60 °C, to 391 at 120 °C, and B changed from 272 to 174, respectively. This indicated that the stability of A was worse with the increasing ATF temperature, but the stability of B was better. As can be seen from Figure 10, the friction torque curves of A and B gradually coincided with the increase in temperature. The difference in friction torque between A and B was most obvious at low temperatures, such as 60 °C.

**Figure 11:**
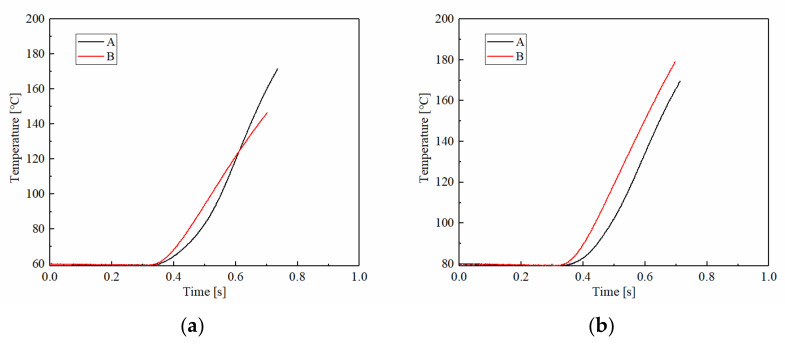

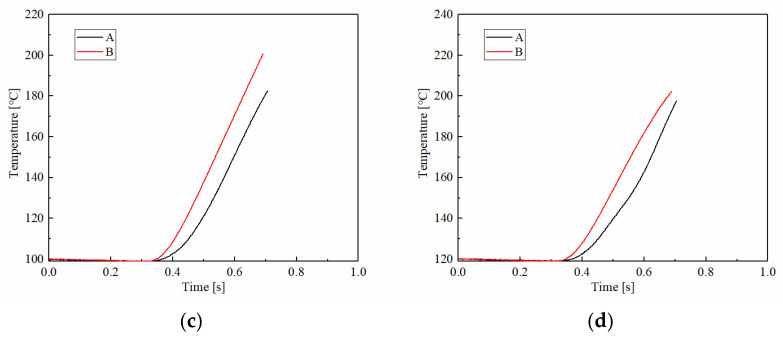
The temperature changes of samples A and B at different ATF temperatures: (**a**) 60 °C; (**b**) 80 °C; (**c**) 100 °C; (**d**) 120 °C.

The temperature vibrations in different ATF temperatures are shown in Figure 11. The temperature change was different to the applied pressure and rotating speed conditions. The temperature of A was lower at first and then higher than B at 60 °C. It can be seen that the slope of the temperature curve remains constant, which means the rising rate of B was relatively stable. As the temperature increased, the rising rate and maximum temperature of B were both slightly higher than A.

#### 3.1.4. The Stability of Friction Performance

**Figure 12:**
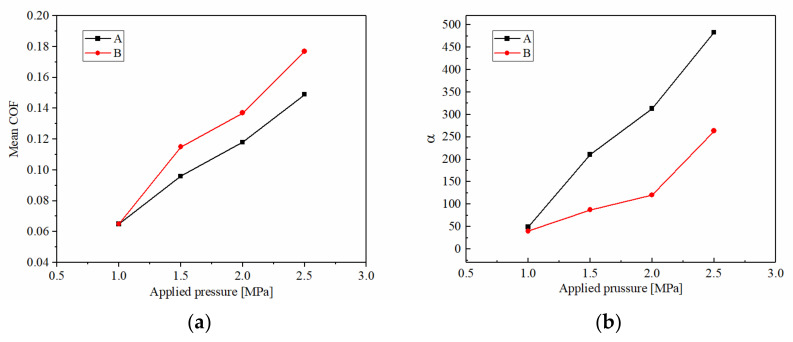

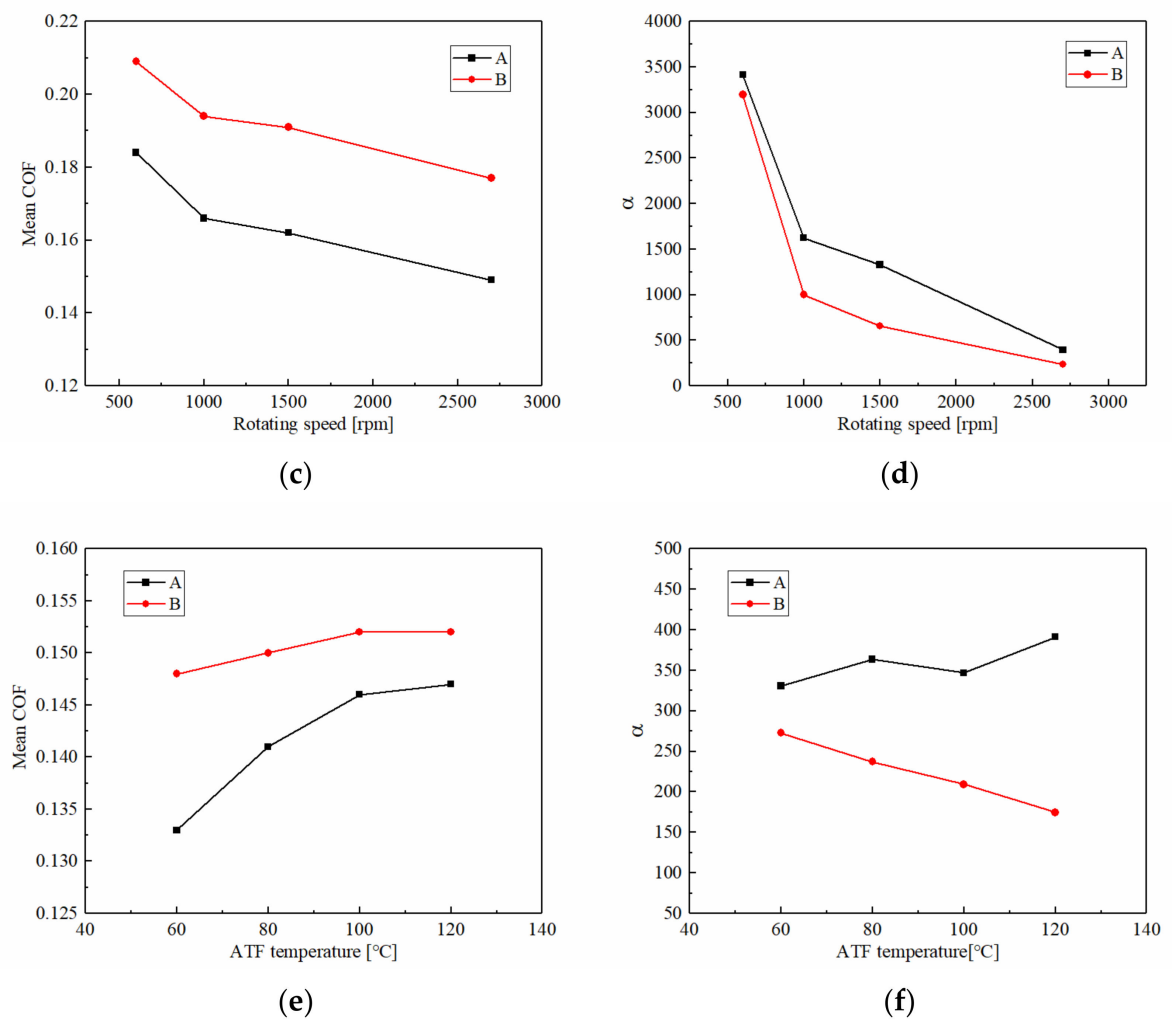
Mean COF and variable coefficient at different applied pressures (**a**,**b**), at different rotating speeds (**c**,**d**), and at different ATF temperatures (**e**,**f**).

Changes in friction performance are closely related to the COF. Mean COF is calculated by friction torque, and the variation trend of the instantaneous COF is described by the variable coefficient (α). Figure 12 depicts the mean COF and α of A and B under different operating conditions. As we can see, the mean COF increased significantly with the applied pressure, but the increasing value of B was significantly higher than A. The mean COF of A increased from 0.065 at 1 MPa to 0.149 at 2.5 MPa, while B increased from 0.065 to 0.177, respectively. Meanwhile, the α of A increased from 47.91 to 483.62, and B increased from 38.18 to 264.62. It showed that under the applied pressure conditions, the mean COF of B was higher and the friction performance was more stable. However, the mean COFs and stabilities of A and B were both increased with the applied pressure.

Under the rotating speed conditions, the situation was the opposite. As the rotating speed increased, the mean COFs and stabilities of A and B significantly decreased with rotating speed. The mean COF of A dropped from 0.183 at 600 rpm to 0.149 at 2700 rpm, and B decreased from 0.208 to 0.177, respectively. The magnitude of the drop was basically the same for both A and B, but the mean COF of B was, overall, higher than A. At the same time, the α of A and B both decreased with rotating speed, but the value of B was lower. It showed that the friction stability of B was also higher than A under the rotating speed conditions.

With the ATF temperature increasing, the mean COF of both A and B clearly increased, but the magnitude of A was significantly higher than B, from 0.132 at 60 °C to 0.147 at 120 °C, but the values were always lower than B which changed from 0.148 to 0.152, respectively. The α of A and B were the opposite, as the value of A increased significantly with the ATF temperature, but B decreased. In total, the friction stability of B was better than A under temperature conditions.

### 3.2. Wear Performance

#### 3.2.1. Surface Topography

**Figure 13:**
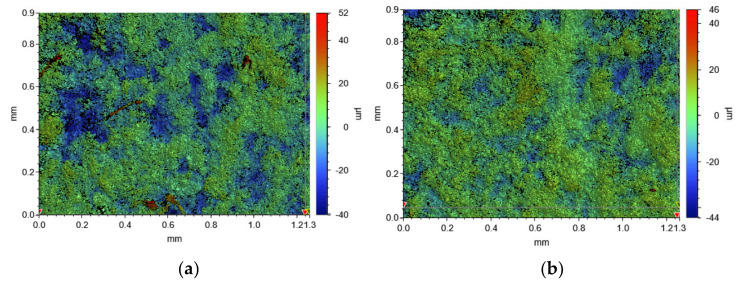
White light interferogram of A (**a**) and B (**b**).

The overall friction and wear performance of paper-based friction material is dependent on the fabrication method, proportions of the raw materials, and topography of the friction surface. Due to the different properties of the reinforcing fibers, the surface morphologies of the two materials were also different. The specific features of A and B are shown in Figure 13; we can see that the surface of A has more concave parts (blue area), and the prominent peaks are also more obvious (red area); while the surface of B is relatively flat, with very few peaks and concave areas.

**Figure 14:**
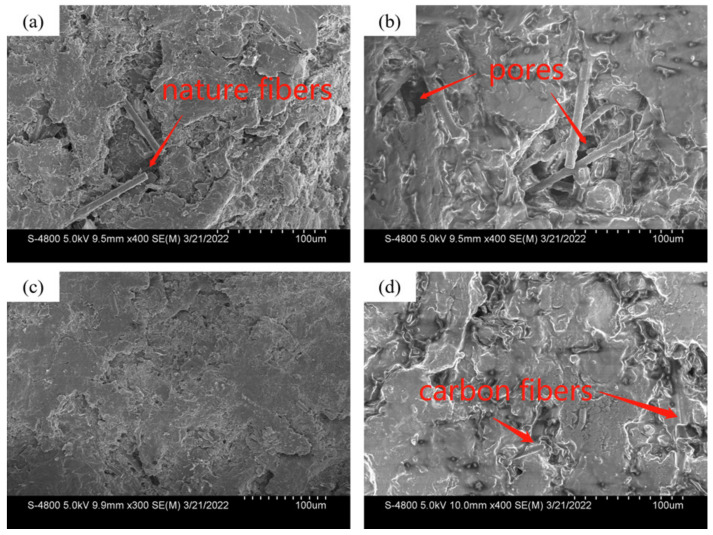
Topography of initial surfaces A (**a**) and B (**c**), and tested surfaces A (**b**) and B (**d**).

However, it is difficult to observe how the carbon fibers are distributed and arranged in the white light interference image. As shown in Figure 14, the photos obtained using the SEM demonstrate the distribution of fibers and their microscopic characteristics in different friction materials. The fibers in A, shown in Figure 14a, can obviously be obtained on the surface, and the fiber crossing is prone to forming pores. Therefore, the actual contact area is reduced, which also explains the main reason for their reduced friction torque. The distribution of short carbon fibers in B is uniform and cross-distributed, as shown in Figure 14c, and it is difficult to see the carbon fibers on the initial surface. However, with the process of friction, the wear leads to the peeling of the surface filler, making the PAN chopped carbon fibers visible, as shown in Figure 14d. It can be seen in Figure 14d, that the tested surface of B has few pores and is shallower than A. The PAN chopped carbon fibers are evenly distributed in the resin matrix, and it is not easy to form agglomeration, which leads to fewer pores on the material surface, and the shallow depth and fewer number of pores increase the contact area so that it can improve the higher friction torque in the process of various operating conditions.

#### 3.2.2. Variation in Thickness

**Figure 15:**
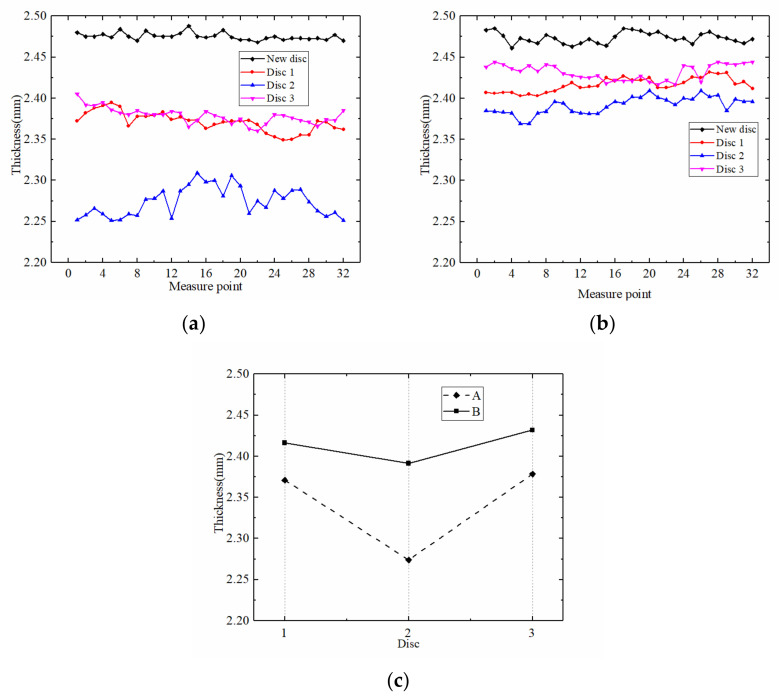
The thickness of the friction disc after the test: (**a**) thickness of different discs in A; (**b**) thickness of different discs in B; (**c**) mean thickness of A and B.

Thickness variation is a good indicator of the wear process. Figure 15a,b present the thickness variations of A and B in three discs. For the initial friction disc, the friction core and friction lining thicknesses were 1.5 mm and 0.5 mm, respectively. The actual average thickness of friction disc was around 2.481 mm. After the experiments, there was a significant difference in the thickness of the friction discs with different axial positions. Figure 15c shows the average thickness of the friction disc in each case. More exactly, in these three cases, the thickness of disc 2 was the smallest, indicating that the thermal load of the friction disc in the axial middle position was the largest, which resulted in the most serious wear performance. Thus, the conclusion can be drawn that the friction discs in case 2 suffered the most serious wear, followed by cases 3 and 1. In addition, the wear degree of B was significantly lower than A. Even the mean thickness of case 2, which had the highest wear degree, was 2.391 mm after the test, which was 0.119 mm higher than A. The thickness values of case 1 and case 3 in B were also higher than A, which showed that the wear resistance of B was better than A. This provides a basis for the application of B in harsh environments.

## 4. Conclusions

From the above research, the following conclusions can be drawn:

- Carbon fiber reinforced friction material can provide a higher friction torque and a lower temperature rise rate under the conditions of changing applied pressure and rotating speed. This provides a basis for the use of friction discs under high-speed and high-power density;
- The excellent thermal conductivity of carbon fiber results in the higher stability of COF, and the change of α decreases significantly. As the pressure increases from 1 MPa to 2.5, the friction torque of carbon fiber reinforced friction materials increases by 400%, and the variable coefficient (α) only rises from 38.18 to 264.62, with the applied pressure increasing from 1 MPa to 2.5 MPa;
- Due to the good dispersion and excellent mechanical properties of PAN chopped carbon fibers, fewer pores form on the initial surface, which improves the high wear resistance, especially in the inter-media disc.

## Figures and Tables

**Table 1:** Experimental operating conditions of the SAE#2 test.

Factors	Applied Pressure (MPa)	Rotating Speed (rpm)	ATF Temperature (°C)
Part 1	1, 1.5, 2, 2.5	2700	100
Part 2	2.5	600, 1000, 1500, 2700	100
Part 3	2	2700	60, 80, 100, 120

## Data Availability

Not applicable.

## References

[1] Wang Z.W., Liu C.S. (2001). Analysis on the friction material of the friction clutch and brake. Zhongguo Gonglu Xuebao China J. Highw. Transp..

[2] Ost W., De Baets P. (2003). The tribological behaviour of paper friction plates for wet clutch applications investigated on SAE II and pin-on-disc test rigs. TriboTest.

[3] Yu L., Ma B., Chen M., Li H., Ma C., Liu J. (2019). Comparison of the friction and wear characteristics between copper and paper based friction materials. Materials.

[4] Iqbal S., Al-Bender F., Ompusunggu A.P., Pluymers B., Desmet W. (2015). Modeling and analysis of wet friction clutch engagement dynamics. Mech. Syst. Signal Process..

[5] Pradeepkumar C., Karthikeyan S., Rajini N. (2021). Materials Today: Proceedings A short review on the effect of transfer layer on tribological study of composite materials. Mater. Today Proc..

[6] He-yun B., Wei H., Feng-xia L. (2021). Investigation of engagement characteristics of a multi-disc wet friction clutch. Tribol. Int..

[7] Shaffer S.J., Freshly T.B., Papanicolaou S.E. (2018). Benchtop screening of wet clutch materials. Tribol. Int..

[8] Ramesh M.R., Ravindra K.A., Ashok B., Kannan C. (2021). Optimizing thermal performance of a dry rigid clutch by varying groove pattern and friction material. Mater. Today Proc..

[9] Neupert T., Bartel D. (2021). Measurement of pressure distribution and hydrodynamic axial forces of wet clutch discs. Tribol. Int..

[10] Wang B., Yan L., Kasal B. (2022). A review of coir fibre and coir fibre reinforced cement-based composite materials (2000–2021). J. Clean. Prod..

[11] Kurdi A., Alhazmi N., Alhazmi H., Tabbakh T. (2020). Practice of simulation and life cycle assessment in tribology—A review. Materials.

[12] Cho H.R., Je Y., Chung K.H. (2018). Assessment of Wear Characteristics of Paper-Based Wet Friction Materials. Int. J. Precis. Eng. Manuf..

[13] Lu J., Li Y., Wang Y., Fu Y. (2018). Effect of pre-impregnated organosilicon layer on friction and wear properties of paper-based friction materials. Wear.

[14] Li M., Khonsari M.M., McCarthy D.M.C., Lundin J. (2015). On the wear prediction of the paper-based friction materialin a wet clutch. Wear.

[15] Yu L., Ma B., Chen M., Xue J., Zhao P. (2020). Variation mechanism of the friction torque in a Cu-based wet clutch affected by operating parameters. Tribol. Int..

[16] Hsia F.C., Elam F.M., Bonn D., Weber B., Franklin S.E. (2021). Tracing single asperity wear in relation to macroscale friction during running-in. Tribol. Int..

[17] Ho S.C., Lin J.H.C., Ju C.P. (2005). Effect of fiber addition on mechanical and tribological properties of a copper/phenolic-based friction material. Wear.

[18] Kumar N., Singh A., Singh S., Singh J.I.P., Kumar S. (2021). Napier natural fibre used as reinforcement polymer composite: As asbestos free brake friction material. Mater. Today Proc..

[19] Kumar S., Manna A., Dang R. (2022). A review on applications of natural Fiber-Reinforced composites (NFRCs). Mater. Today Proc..

[20] Prabhakar C.G., Anand Babu K., Kataraki P.S., Reddy S. (2021). A review on natural fibers and mechanical properties of banyan and banana fibers composites. Mater. Today Proc..

[21] Mohankumar D., Rajeshkumar L., Muthukumaran N., Ramesh M., Aravinth P., Anith R., Balaji S.V. (2022). Effect of fiber orientation on tribological behaviour of *Typha angustifolia* natural fiber reinforced composites. Mater. Today Proc..

[22] Singh T. (2021). Optimum design based on fabricated natural fiber reinforced automotive brake friction composites using hybrid CRITIC-MEW approach. J. Mater. Res. Technol..

[23] Gweon J.H., Joo B.S., Jang H. (2016). The effect of short glass fiber dispersion on the friction and vibration of brake friction materials. Wear.

[24] Zhang X., Li K.Z., Li H.J., Fu Y.W., Fei J. (2014). Tribological and mechanical properties of glass fiber reinforced paper-based composite friction material. Tribol. Int..

[25] Duan J., Zhang M., Chen P., Li Z., Pang L., Xiao P., Li Y. (2021). Tribological behavior and applications of carbon fiber reinforced ceramic composites as high-performance frictional materials. Ceram. Int..

[26] Li M., Khonsari M.M., McCarthy D.M.C., Lundin J., Fei J., Luo W., Huang J.F., Ouyang H., Xu Z., Yao C. (2021). Effect of carbon fiber content on the friction and wear performance of paper-based friction materials. Tribol. Int..

[27] Saindane U.V., Soni S., Menghani J.V. (2021). Studies on mechanical properties of brake friction materials derived from carbon fibres reinforced polymer composite. Mater. Today Proc..

[28] Fei J., Li H.J., Fu Y.W., Qi L.H., Zhang Y.L. (2010). Effect of phenolic resin content on performance of carbon fiber reinforced paper-based friction material. Wear.

[29] Rehman Z.U., Ahn B.H., Jeong Y.S., Song J.I., Koo B.H. (2016). The influence of various additives on the properties of PEO coatings formed on AZ31 Mg Alloy. Surf. Rev. Lett..

